# Microbiome and Asthma: Microbial Dysbiosis and the Origins, Phenotypes, Persistence, and Severity of Asthma

**DOI:** 10.3390/nu15030486

**Published:** 2023-01-17

**Authors:** José Valverde-Molina, Luis García-Marcos

**Affiliations:** 1 Department of Paediatrics, Santa Lucía General University Hospital, 30202 Cartagena, Spain; 2 Paediatric Allergy and Pulmonology Units, Virgen de la Arrixaca University Children’s Hospital, University of Murcia and IMIB Biomedical Research Institute, 20120 Murcia, Spain

**Keywords:** asthma, microbiome, microbiota, dysbiosis

## Abstract

The importance of the microbiome, and of the gut-lung axis in the origin and persistence of asthma, is an ongoing field of investigation. The process of microbial colonisation in the first three years of life is fundamental for health, with the first hundred days of life being critical. Different factors are associated with early microbial dysbiosis, such as caesarean delivery, artificial lactation and antibiotic therapy, among others. Longitudinal cohort studies on gut and airway microbiome in children have found an association between microbial dysbiosis and asthma at later ages of life. A low α-diversity and relative abundance of certain commensal gut bacterial genera in the first year of life are associated with the development of asthma. Gut microbial dysbiosis, with a lower abundance of *Phylum Firmicutes*, could be related with increased risk of asthma. Upper airway microbial dysbiosis, especially early colonisation by *Moraxella* spp., is associated with recurrent viral infections and the development of asthma. Moreover, the bacteria in the respiratory system produce metabolites that may modify the inception of asthma and is progression. The role of the lung microbiome in asthma development has yet to be fully elucidated. Nevertheless, the most consistent finding in studies on lung microbiome is the increased bacterial load and the predominance of proteobacteria, especially *Haemophilus* spp. and *Moraxella catarrhalis*. In this review we shall update the knowledge on the association between microbial dysbiosis and the origins of asthma, as well as its persistence, phenotypes, and severity.

## 1. Introduction

Asthma is a chronic disease, product of the interaction between genes and the environment. Its physiopathology is complex, presenting several phenotypes: T2 (allergic and non-allergic eosinophilic) and not T2 (non-eosinophilic). Although there is heterogeneity in their origins [1] there is ample evidence of the influence of environmental exposure, above all microbial, during infancy [2,3].

At the end of the 1980s, Strachan postulated the “hygiene hypothesis” [4]. Since then, different studies have found an association between early exposure to environmental situations with an elevated bacterial load (day-care, dogs in the home, farms) and decreased development of asthma and allergies. There is evidence of environmental factors that promote asthma and allergies [5,6,7,8]. This “farm effect” would provide immunomodulatory stimuli in sufficient amount and of sufficient quality to promote immunisation, but with an individual variability, which may be due to multiple factors, such as the age of exposure, the present of allergic sensitisation, the host’s genotype and interactions [9]. Recently, as an extension to this hypothesis, the “microflora hypothesis” has appeared, indicating that the alteration of the bacteria that reside in the human gut in the first stages of life, due to antibiotics use, infections, or diet, destroys the normal microbial mechanisms that promote immunological tolerance and make the immune system move toward a state that promotes allergic disease and asthma [10].

We understand *microbiota* to refer to the microbes that colonise the skin and mucosa of the different body surfaces, and *microbiome* to be the sum of these microbes, their genomic elements, and their interactions with a certain ecological niche. Both terms are used in the literature interchangeably [11].

Measuring microbiota implies collecting stool samples or samples from the airways which can be characterised by its diversity and profusion. The diversity designates the number of “diverse” (hence the name) taxa within a community. The total number of taxa in the sample is the α-diversity refers to, whilst diverse in composition between samples is called β-diversity. Recently, artificial intelligence and genomic sequencing have moved forward considerably. The platforms that are available enable the simultaneous sequencing of the majority or all of the genetic material present in the samples. This enables deeper and more directed exploration into the microbiota’s composition and the functional dynamics of the community. One widely used genomic sequencing technique is the focus on amplification of the ribosomal 16S rRNA gene, highly preserved in all bacteria species. The technique allows resolution at genus level. The sequencing of all the DNA present in an unselected sample is named “Shotgun” metagenomic sequencing. This new sequencing generation allows better knowledge on the normal development and functions of microbiota [12,13,14].

Colonisation with microorganisms begins at birth, representing an adjustment period in the metabolic and immunological development of the infant to their surroundings [15], where epigenetic changes are very important for health. These changes are brought on by environmental factors that influence the genes during development [16]. There is a critical window in the early stages of life when the alteration of this microbiota may favour the development of diseases in later stages [17]. This process of normal bacterial colonisation is known as *symbiosis*, while *dysbiosis* is understood to refer to the disruption of this colonisation in the uterus and during infancy, which would participate in the expression of diseases mediated by metabolic and/or immune processes. Dysbiosis may be due to a loss in beneficial microorganisms, expansion of pathobionts or loss of microbial diversity [18], and may alter the immunological development in tissues in the mucosa and provoke greater susceptibility to asthma.

Different reviews have analysed the association between the microbiome and asthma [9,12,13,19,20,21,22]. This review intends to update the evidence on the relationship between microbial dysbiosis and the origin, progression, and severity of asthma.

## 2. Gut and Airway Microbiome: Gut-Lung Axis

### 2.1. Gut Microbiome

In the last decades, multiomics techniques (metatranscriptomics, metagenomics, metabolomics and metaproteomics) have enabled us to know more about the complex ecosystem of gut microbiota. Gut microbiota contains between 1000 and 5000 species of bacteria. However, each person only has around 160. Moreover, gut microbiota comprises of around 3.3 million genes. In healthy individuals it is composed mainly of the phyla: *Firmicutes*, *Bacteroidetes*, *Proteobacteria* and *Actinobacteria*, above all *Firmicutes* and *Bacteroidetes*, and the class *Clostridia* dominates the sequences of *Firmicutes*. Nevertheless, microbiome includes not only bacteria, but also other organisms [10,23,24].

The gut microbiome composition is dynamic, especially in the first years of life, when it diversifies rapidly prior to stabilising, with respect to the distribution of the dominant bacteria phylum, at the age of three years [25]. 

Gut colonisation begins in the uterus [26] with a limited number of bacteria, including species of *Lactobacillus*, *Staphylococcus* and *Micrococcus* which would contribute to priming the immune cells and to the development of populations of memory T-cells with experience in bacterial antigens [27]. The placenta hosts a very specific microbiota which includes non-pathogen commensal microbiota resembling the mother’s oral microbiota, which indicates that the placenta microbiota could be established by haematogenic propagation from the oral microbiota. Furthermore, microbial DNA has been found in meconium with considerable similarity between the microbes in meconium and those in the amniotic liquid, which would suppose the existence of a “transplacental colonisation route” [10]. Subsequently, the exposure to and ingestion of microbes occurs when crossing the birth canal. 

The bacterial composition of the newborn includes *Enterococcus*, *Escherichia*, *Streptococcus* and *Rhotia*, which suggests a relatively aerobic gut environment. From 2–4 months of life almost all infants are colonised by *Enterobacteriaceae*, *Bifidobacteriaceae* and *Clostridiaceae*, indicating a reduced concentration of oxygen and use of lactic acid, with a continual decrease until 18 months, following a model known as “maturation of healthy microbiota” [26]. The gut microbiome is very changing during the first three years after birth, with a lower diversity index and greater interindividual variability [17]. Diversity gradually increases over time, and the development to an adult-type microbiota composition, with elevated levels of *Firmicutes* and *Bacteroidetes* and lower levels of *Actinobacteria*, *Proteobacteria* and *Verrucomicrobia*, is still ongoing at the age of three years [26].

#### 2.1.1. Factors That Affect the Microbiome

The factors that influence microbial colonisation and composition may be prenatal, perinatal, and postnatal [2,17,28].

##### Maternal Factors during Pregnancy

Genetics, the environment, and diet affect the gut and vaginal microbiota of the pregnant woman [29]. A diet rich in fats has been related with meconium rich in *Enterococcus* and low in *Bacteroides* until six weeks of life [30], with extraction of energy by degradation of polysaccharides to short chain fatty acids (SCFA), which promote adequate immunity of the gut mucosa through the stimulation of CD4 lymphocytes [26].

Placental and maternal microbiota are closely, being the most abundant species *Prevotella* and *Neisseria*, and *E. coli*. This may be modified by nutritional status and antibiotic use [26,31], which may cause “uterus dysbiosis”.

##### Mode of Delivery

He largest entry of microbes into the foetus occurs during birth, following the breaking of membranes and the ascent of microbes along the birth canal. The concept of “verticality” is important [32].

Models of early colonisation differ depending on the mode of delivery. During a vaginal delivery the neonate enters into contact with maternal vaginal/colon microbiota and ingests it, but during a caesarean delivery the first contact is with the microbiota of the mother’s skin, which implies a lower diversity in the development of the colonisation [33,34].

One systematic review revealed a greater abundance of certain gut bacteria following vaginal delivery with respect to caesarean delivery, above all of the genera *Actinobacteria*, *Bacteroides* and *Bifidobacterium* [35]. Stokholm et al. recently found that intestinal tract colonisation of the newborn by *Escherichia coli* at the age of one week was associated with vaginal delivery, whilst caesarean delivery was associated to colonisation by *Citrobacter freundii*, *Clostridium* species, *Enterobacter cloacae*, *Enterococcus faeccus cloacae*, *Enterococcus faecalis*, *Klebsiella oxytoca*, *Klebsiella pneumoniae* and *Staphylococcus aureus* [36].

##### Prematurity

Rapid passage through the birth canal decreases the possibility of ingesting the maternal vaginal/colon microbiota, thus the diversity is lower, with an increase in proteobacteria [16,26].

##### Type of Lactation

An optimal colonisation requires exclusive breast milk (BM) for the first 4–6 months of life [33]. IgA-type antibodies in breast milk protect against pathogens and other antimicrobial factors. It also hosts its own microbes, whose composition is independent of genetic or environmental factors, which are transmitted together with non-digestible human milk oligosaccharide (HMO) complexes, a type of prebiotics that promote the proliferation of beneficial gut microbes and prevent colonisation by pathogens, as well as stimulating the development of immune factors to reach adequate immune homeostasis. Moreover, microbes are also transferred from the mother’s skin during suction, and also from the gut. They can reach the mammary glands via dendritic cells and macrophages [17]. This is known as the “entero-mammary pathway hypothesis”, and it is calculated that each cubic centimetre of breast milk may contain up to 10^3^ microorganisms, which represents a “natural inoculum” [37,38].

With BM, *Bifidobacteria* and *Lactobacillus* dominate, and stimulate the endogenic production of slgA, and the activation of T regulatory and anti-inflammatory cells [16], which influences a shift from an intrauterine Th2-high response to a more balanced Th1/Th2 response and prevents the expression of allergic asthma [33].

Children with genetic predisposition (17q21) could benefit from the protective effect of BM against early respiratory infection and the secondary prevention of asthma [39]. Breast milk’s microbiome composition differs between those born at term and premature [40]. 

In formula-fed infants these BM microbes are lacking, and there are higher proportions of *Bacteroides*, *Clostridium*, *Streptococcus*, *Enterobacteria* and *Veillonella* spp. [17,41]. 

It has been well documented that the gut microbiota of infants fed on formula milk show a broader diversity than those fed on BM, which, during the first week of life, tends to be dominated by *Bifidobacteria*, with a concomitant decrease in members of the *Enterobacteriaceae* family. Therefore, during their first six months, infants can benefit from exclusive breastfeeding [10].

##### Early Contact with a Greater Number of Persons

Different studies have found an increase in bacterial diversity and richness in the gut microbiome, and a greater probability of having nasal microbiota dominated by *Moraxella* and faeces by *Bifidobacterium* in contact with elder siblings [2].

##### Antibiotic Use

Early exposure to antibiotics may alter the ecology of the gut microbiome and alter its abundance and diversity, which could suppose an overgrowth of other phyla of bacteria [17]. Analysis of the faeces of newborn infants, even premature infants, who received antibiotics in the first days of life exhibited a lower abundance and diversity in *Bifidobacterium* colonisation and an increased abundance of *Enterobacter*, *Escherichica* and *Enteroccocus* [10,42]. This has been recovered at one year of life, although repeated exposure during the first year could result in a less stable microbial community and a fall in its diversity that lasts until three years of life [26]. In addition, in animal models, antibiotics can terminate with the gut microbiota and, subsequently, retard the development of the immune system, which could be related with the inception of hyperreactivity of the respiratory pathways in susceptible infants, and therefore increase the risk of allergic diseases such as asthma [10].

##### Introduction of Solid Foods and Diet

Complementary feeding seems to be crucial to the differential growth of the gut microbiota at early ages. Short-chain fatty acids (butyrate, acetate and propionate), products of microbial fermentation, are crucial energy sources for gut cells [43]. In children from a rural population in Africa where the diet was low in animal fats and proteins but rich in vegetable fibre and polysaccharides, *actinobacteria* and *Bacteroidetes* predominated and they had more butyric acid, a reversible situation after westernising the diet, elevated in proteins from animals, sugars and fat, and low in fibre [44,45].

##### Environmental and Geographical Factors

The setting, which includes mainly the family lifestyle, the family size and its structure, close siblings, the order of birth and the geographical location, has also been considered a factor that may affect the infant gut microbiota [10].

### 2.2. Microbiome of the Airways

The Human Microbiome project, established in 2007 to study the function and diversity of the microbiome did not incorporate samples of the airways, assuming them to be sterile [46]. However, the application of “next-generation sequencing” based on the sequencing and analysis of the 16S rRNA gene for studying the airway microbial composition in healthy persons has changed the paradigm regarding its sterility. Microbiome study can be performed by means of samples from the upper (nose, nasopharynges, oropharynges and saliva) and lower (sputum, bronchial aspiration or bronchoalveolar lavage) airway, with the latter being the most difficult to obtain in the paediatric population. In this case, studying both is relevant [13,47].

We currently know that the airway is colonised by niche-specific bacterial communities, with a density up to 10^3^–10^4^ times lower in the lower airway with respect to the upper airway, and as with the gut microbiome, its colonisation also begins at birth and becomes stable in the first years of life [12,47].

At nasopharyngeal level, following their stabilisation, the predominant bacteria communities are associated with *Dolosigranulum* and *Moraxella* spp. They are modulated by day-care attendance, viral infections and antibiotic treatments, amongst other factors [12,48]. Six phyla are predominately detected in the lungs of healthy persons: *Firmicutes*, *Proteobacteria*, *Bacteroidetes*, *Eusobacteria*, *Acidobacteria* and *Actinobacteria*, the diversity increases in asthma with the *Proteobacteria* phyla [11,49,50].

Colonisation of the lower airway is closely related to its anatomy and function, with migration from the upper airway being the main source in healthy individuals [51]. The lung microbiome would be determined by the equilibrium between microbe migration by micro-aspirations, inhalation and dispersion by the mucosa and the elimination through coughing, mucociliary clearing, innate and adaptive host defences, and the existing conditions for bacterial growth such as the pH, the oxygen pressure, the nutrient concentration, and the presence of inflammatory cells, with biogeographical variations in the microbiome composition. A low diversity and a continual renewal with a low replication rate are observed in healthy individuals. A disbalance, due to the presence of conditions that favour the replication or persistence of certain species, could favour the development of asthma [12,19]. Several factors (internal and external) may influence the lung microbiome composition. Nutrition, age, lifestyle, tobacco smoke or pollution, inherited genes and underlying conditions may influence the type and number of lung microbiota, which may cause dysbiosis [52]. There is an overlap in the characteristics of the microbiome between the respiratory pathways in healthy children and adults where *Bacteroidetes* predominates, as opposed to adult and paediatric asthma patients where there is increased *Proteobacteria* [49].

### 2.3. The Gut-Lung Axis

Recently Schudijt et al. proposed the hypothesis of the existence of a gut-lung axis. Although the exact mechanisms by which this axis would activate the innate immune system are not wholly known, there is evidence of possible interactions between the gut and lung mucosa [53]. It is likely that this axis is important to maintain normal microbiota and influence the immune response in both compartments and maintain homeostasis [54]. Several studies have shown that the gut microbiome modulates the functioning of Treg lymphocytes producing local and systemic mediators that influence the development of asthma mediated by the gut-lung axis [55]. Moreover, bioactive and bacterial ligands and metabolites from the gut could enter into circulation to interfere with the migration of immune cells in the airway [14].

Gut microbes may influence the immune function of the lung through different mechanisms. One of these is the connection by means of molecular shapes associated to pathogens, such as lipopolysaccharides, which may stimulate *toll-like* receptors and trigger genes that regulate inflammation and innate immune system responses. Additionally, it would provoke phenotype changes in the dendritic cells and migration to the mesenteric lymph nodes to promote T lymphocyte priming. In these lymph nodes the T cells acquire localisation molecules that start the migration to the respiratory mucosa, where they bring about changes in the anti-inflammatory response. Another mechanism is through the metabolites such as SCFA, which are produced by bacteria after the fermentation of carbohydrates. SCFA modify the genetic expression by means of inhibiting histone deacetylases, methylation, cytokine and chemokine production, cell differentiation, proliferation, and apoptosis. Finally, an effect through epigenetic changes is possible [9,43,56,57]. The interaction between alveolar macrophages, Treg cells and dendritic cells in the respiratory pathways is the most important to maintain its immunological tone [52].

## 3. Microbiome and Immune System

As mentioned above, proper bacterial colonisation in the first years of life is fundamental for the maturation of the immune system, and the balance between its innate and adaptive responses for adequate immune tolerance and inflammatory reaction [11,29]. The ideal conditions result in a symbiotic relationship between the colonising bacteria and the gut epithelial lymphoid tissues, and the immune and metabolic homeostasis for children [16]. 

Members of the genera *Bifidobacterium*, *Lactobacillus* and *Clostridium* increase the proportion of Treg cells. *Clostridium* stimulate the ILC3s innate lymph cells to produce IL22, which helps to reinforce the epithelial barrier and to reduce its permeability. *Bifidobacteria* and *Lactobacilli* may stimulate metabolic pathways in the dendritic cells and promote the induction of Treg cells. The capsular polysaccharide A of *Bacteroides fragilis* has been shown to have the capacity to interact directly with dendritic plasmacytoid cells in mice and promote the secretion of IL10 by the T CD4+ cells. Moreover, an exopolysaccharide of *Bifidobacterium longum* seem to supress the Th17 response in the gut and lung [11,58].

However, bacterial colonisation does not influence immune development alone, its metabolites and secreted products following the interaction with the gut may also help to modulate the adaptive function. Short-chain fatty acids, product of the fermentation of dietary fibre, are ligands for GPR43 expressed for the Treg cells and stimulate their expansion and immunosuppressor properties, such as the production of IL10, thereby controlling pro-inflammatory responses in the gut [59]. They also influence epithelial cell integrity and promote epigenetic changes through their inhibition of histone deacetylases [43,60], protecting against allergen-induced airway inflammation [61]. This occurs with the increase in acetate, butyrate and propionic acid production [62,63]. Other metabolites, such as p-cresol sulphate, a derivative of L-tyrosine, which reduces the production of CCL20 (a chemoattractant lymphocyte) by the respiratory epithelial cells derived from the microbiome, protect against allergic inflammation of the airway. In the colon, commensal bacteria such as *Bacteroides fragilis* produce anti-inflammatory polysaccharide A-like products that decrease the production of IL-17. However, other metabolites such as oxylipins derived from gut bacteria that promote allergic inflammation by diminishing the frequency and productivity of IL-10 by the lung Treg population may increase the risk of asthma [20]. 

## 4. Microbiome and Asthma in an Animal Model

Different studies performed on germ-free mice support the role of the microbiome in the development of airway allergic inflammation and asthma. Herbst et al. observed the existence of Th2-type inflammation and airway hyperresponsiveness following stimulation with ovalbumin in germ-free mice, but not in mice colonised with commensal microbes. After cohabiting for three weeks, both types of mice showed reduced levels of allergic inflammation, which indicates that gut and airway colonisation with commensal microbes had a protective effect [64]. Gollwitzer et al. analysed the susceptibility to induce allergic inflammation by house-dust mites in mice at 3, 15, and 60 days of life, stimulating the conditions of progressive colonisation of the airway. The neonatal mice developed airway eosinophilia, liberation of Th2-type cytokines and bronchial hyperresponsiveness after exposure to the mites compared with older mice. The protective effect in these older mice was due to the progressive microbial colonisation of the airway and the shift from *Gammaproteobacteria* and *Firmicutes* to *Bacteroidetes*. Lung microbiota maturation was associated with the appearance of T Helios regulatory cells – dependent on PD-L1 [65]. Stein et al. found that the intranasal instillation of dust from Amish homes, but not from those of Hutterites, reduced the airway allergic inflammation induced by ovalbumin in mice via Myd88 and Trif-dependent mechanisms. The dust from Amish homes had different bacterial populations, especially *Bartonellaceae*, and higher levels of endotoxins compared to the dust from Hutterite homes [66]. Gut colonisation with *Bifidobacterium infantis* makes atopic asthma symptoms to lower in a mouse model: this is due to the regulation of the balance between Th1 and Th2 responses [67].

## 5. Evidence of the Association between Microbial Dysbiosis and Asthma

### 5.1. Microbial Dysbiosis and Asthma Origin 

The pathogenesis of infant asthma could be related to factors that, during the foetal period and the first years of life, provoke an alteration in the microbiome diversity at gut and airway level, with the consequent alteration in the development, maintenance and control of the immune system [19]. 

The capacity of immune system cells to produce inflammatory cytokines and establish a memory response to microbes is already evident in the foetal gut from the second semester of pregnancy, being partially dependent on the maternal microbiome and relevant for the development of asthma [27]. 

**Figure 1:**
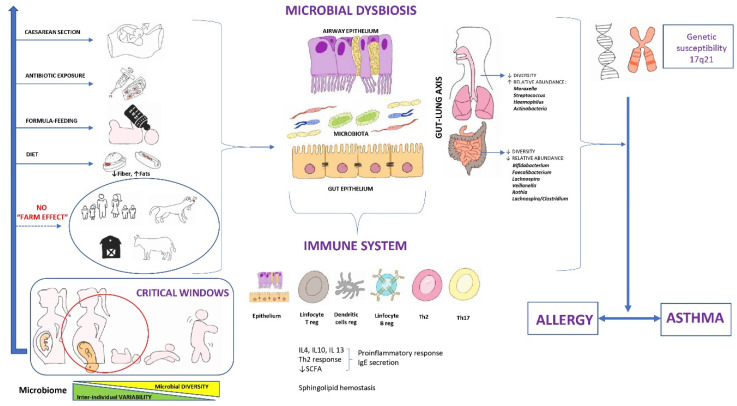
Microbial dysbiosis as an early origin of asthma. Modified and with permission from Valverde-Molina J [22].

In postnatal life, the perturbation of the microbiome together with metabolic dysfunction increases the risk of developing allergy and asthma in infancy, an association that remains evident in pre-school aged children. This fact, together with the knowledge that microbes adjust the immune function, indicates that temporal and dynamic microbial-immune interactions are central to the development and chronicity of allergy and asthma [20]. A mechanistic comprehension arises as to how environmental microbial products such as lipopolysaccharide (LPS) and the fall in sphingolipid concentrations associated to variants of the 17q21 gene alter susceptibility to asthma [12,68]. A scheme on the “microbe theory” as the origin of asthma is given in Figure 1.

**Figure 2:**
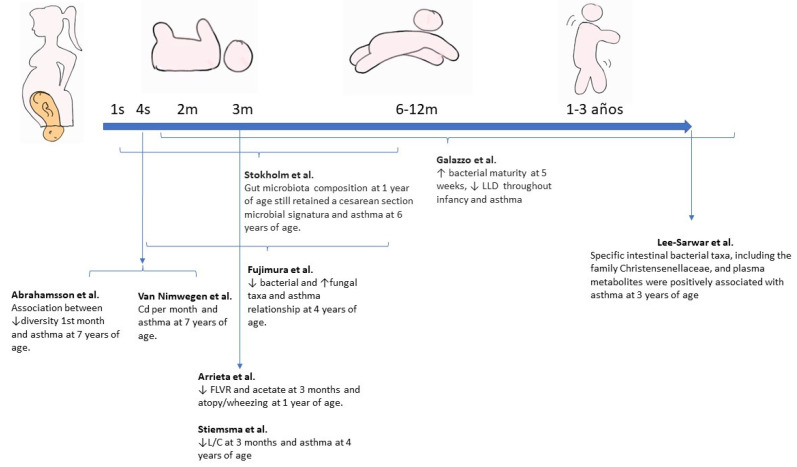
Main studies on intestinal microbiome and its relationship with childhood asthma. Modified and with permission from Valverde-Molina J. [22]. NF: Nasopharyngeal; Sp: *Streptococcus pneumoniae*; Hi: *Haemophilus influenzae*; Mc: *Moraxella catarrhalis*; Cd: *Clostridium difficile*; FLVR: *Faecalibacterium*, *Lachnospira*, *Veillonella*, and *Rothia*; L/C: *Lachnospira*/*Clostridium* ratio; LCC: *Lachnobacterium*, *Lachnospira*, Dialister. Arrieta et al. [71]; Fujimura et al. [73]; Galazzo et al. [78]; Lee-Sarwar et al. [86]; Stiemsma et al. [72]; Stokholm et al. [36]; Van Nimwegen et al. [69].

**Figure 3:**
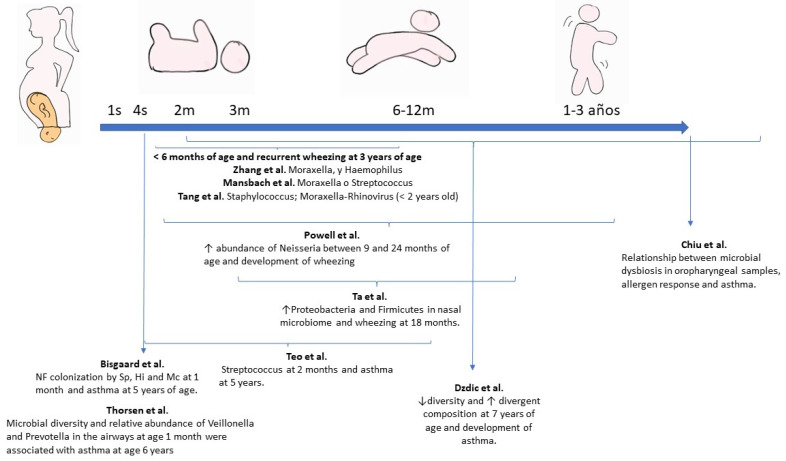
Main studies on respiratory microbiome and risk of asthma in children. Modified and with permission from Valverde-Molina J. [22]. NF: Nasopharyngeal; Sp: *Streptococcus pneumoniae*; Hi: *Haemophilus influenzae*; Mc: *Moraxella catarrhalis*. Bisgaard et al. [74]; Chiu et al. [76]; Dzdic et al. [81]; Mansbach et al. [85]; Powell et al. [83]; Ta et al. [77]; Tang et al. [87].; Teo et al [75,79]; Thorsen et al. [82]; Zhang et al. [84].

Several longitudinal cohort studies have demonstrated the association between dysbiosis during this period and the development of atopy and asthma in later life [36,69,70,71,72,73,74,75,76,77,78,79,80,81,82,83,84,85]. The main studies on microbiota and asthma are summarised in Figure 2 (intestinal microbiome) and Figure 3 (respiratory microbiome).

#### 5.1.1. Gut Microbiome and Asthma Origin

The development of asthma in infancy is closely related to altered infant microbiota, which leads to the loss of the protective effect of a “normal” microbiota [54] and its metabolic activity. Changes in microbiota are more prominent in the first hundred days of life, a period in which the immune system has greater plasticity. It is considered an important critical timeframe [56,88], as well as a window of opportunity for the immune system to be properly educated [89]. 

Dysbiosis may often occur during the initial gut colonisation of the newborn, as a consequence of maternal dysbiosis during pregnancy, caesarean delivery, prematurity or the excessive use of antibiotics perinatally. This, associated with artificial lactation and inadequate stimulus by solid foods, causes a delay in the colonisation period until the age of 4–6 years of life, so the child is more susceptible to infections and immune-system-mediated diseases [28,90].

One meta-analysis showed a 20% increase in the risk of being diagnosed with asthma after a caesarean delivery compared to a vaginal delivery [91]. The lower diversity and low colonisation by *Bacteroides* and *Bifidobacterium* observed in those delivered by caesarean section may result in an inappropriate stimulation of the immune system and in a predisposition for Th2-mediated allergic disease [26]. Epidemiological studies have shown an association between antibiotic consumption in the first year of life and asthma at school age and adolescence [92,93]. Antibiotic use provokes a delay in the microbiota maturation and the depletion of *Lachnospiraceae*, producers of SCFA, which are important in the maturation of immunity by epithelial signalling cells, colon Treg cells and macrophages [28]. Breast milk is very rich in nutrients and substances with a prebiotic function, such as HMO, which favour the growth of *Bifidobacterium* and *Lactobacillus*, and additionally are bacteria contained in BM. These bacteria metabolise the HMO to produce acetate and lactate. Reduced acetate in faeces at three months of life has been associated with allergic diseases at older ages. Moreover, the lactate helps to maintain the gut barrier function by stimulating the proliferation of enterocytes [71]. A Canadian cohort studying 3296 children from birth found that direct breastfeeding provided the greatest protection against the risk of developing asthma, in comparison with artificial lactation or extracted BM [94]. A recent systematic review and meta-analysis revealed evidence of the protective effect of BM against asthma in the 5–18 years age range [95], above all in children breast fed for at least four months [96]. Atmospheric pollution could alter the gut microbiome increasing Bacteroidetes and decreasing Firmicutes, and such changes may be associated to the development of asthma. Furthermore, tobacco smoke exposure both intrauterine and after birth presented an increase in the abundance of *Enterobacteriaceae* that may be associated to a risk of respiratory symptoms in infancy [55].

The role of the gut microbiome and its metabolites in the pathogenesis of asthma has been the subject of study in recent decades.

Van Nimwegen et al., in a prospective cohort in the Netherlands (KOALA Study), analysed gut microbiome, finding an association between colonisation at one month of life by *Clostridium difficile* (phylum *Firmicutes*) species with the presence of wheezing and asthma at the age of 6–7 years [69].

Abrahamsson et al., in a cohort in Sweden, found a relationship between lower diversity in the gut microbiome in the first month of life and the development of asthma at seven years [70]. 

Arrieta et al., in a Canadian cohort (CHILD Study), found that children at risk of asthma had lower relative amounts of bacteria of the genera *Faecalibacterium*, *Lachnospira*, *Veillonella* and *Rothia* at young ages. Additionally, this dysbiosis was accompanied by low levels of faecal acetate and dysregulation of enterohepatic metabolites [71]. Stiemsma et al., in the same cohort, reported that children diagnosed with asthma at four years of age had a gut microbiome at three months of life with a drop in the relative abundance of the genus *Clostridium neonatale*, and concluded that the L/C ratio could be an early biomarker to predict future asthma [72].

Fujimura et al., in a cohort of 308 children in Detroit followed until the age of 48 months of life, found that the newborns who presented lower relative abundance of *Bifidobacteria*, *Akkermansia* and *Faecalibacterium* and relative abundance of fungi of the types of *Candida* and *Rhodotorula* had a greater risk of developing atopy and asthma and allergic diseases [73].

Stokholm et al. carried out a prospective study in 700 children to determine the risk of asthma inception in the first six years of life. Those children who conserved a gut microbial fingerprint at the age of one year were more prone to having asthma at the age of six years [36].

Galazzo et al., in a German cohort of 440 children followed until school age found an association between an increase in the bacteria maturity at five weeks, with a lower abundance of the taxa *Lachnobacterium*, *Lachnospira* and *Dialister* throughout infancy and the risk of developing asthma at older ages [78].

Alcazar et al. published the first systematic review that analysed the association between gut microbiota after birth and respiratory disease in infancy. Studies with >700 participants showed that a higher α-diversity in the first years of life was associated to not having asthma at 5–6 years. In general, there was proof that the low relative abundance of *Bifidobacterium* in faeces collected prior to three months of age was associated with respiratory tract infections at one year of age and with asthma at 4–5 years. Additionally, the low amount of the genera *Faecalibacterium*, *Roseburia* and *Ruminococcus* in stools samples collected at 3–12 months of life was associated with asthma and atopic wheezing at 1–6 years of age [14].

Roduit et al. studied a cohort of children born in rural zones of five European countries (Austria, Finland, France, Germany, and Switzerland), measuring the SCFA in stools of 301 one-year-old children. They found that the presence of high amounts of butyrate and propionate in the stool samples represented a protective effect against sensitisation and development of asthma between the ages of three and six years [97].

Lee-Sarwar et al. carried out an analysis that integrated the diet, microbiome, and the gut and plasma metabolomics in 361 three-year-old infants in the United States. They found negative associations between asthma and gut polyunsaturated fatty acids (PUFAs). Furthermore, both specific gut bacterial taxa such as the *Christensenellaceae* family, and plasmatic metabolites, such as γ-tocopherol/β-tocopherol, associated positively with asthma [86].

The bacterial synthesis of sphingolipids is limited to the members of Bacteroidetes and to certain species of *Proteobacteria*. Studies carried out have demonstrated the immunomodulatory activity of the polysaccharides derived from *B. fragilis* that stimulate T CD4+ cells and correct Th1/ Th2 imbalances. Natural killer cells can be recruited in the gut by these bacterial sphingolipids, which can also increase the proliferation of those subset of T cells, linked to multiple asthma models. Thus, situations in which *Bacterorides* spp. are reduced and consequently the homeostasis of the sphingolipids is altered, as it happens in the neonate microbiome born by caesareans section, there is probably an increased risk of asthma in the later ages [68].

#### 5.1.2. Airway Microbiome and Asthma Origin

Upper airway colonisation begins at birth, with a predominance of *Firmicutes*, *Proteobacteria*, *Actinobacteria* and *Bacteroidetes* phyla being detected in tracheal aspirations within hours after the birth [98].

Bisgaard et al. studying a Danish cohort, analysed samples obtained by means of hypopharyngeal aspiration in 321 asymptomatic infants at one month of life and followed their subsequent evolution, and found that colonisation of the upper respiratory tract at four weeks of life by *S. pneumoniae*, *H. influenzae* and/or *M. catarrhalis* in children with an asthmatic mother was associated to an increased risk of wheezing and asthma at the age of five years and to higher blood IgE eosinophil levels [74].

Teo et al. analysed the nasopharyngeal microbiome in a prospective cohort of 234 infants in Australia several times in their first two years of life. Early colonisation by *Streptococcus*, related with day-care attendance and antibiotic administration, increased the risk of asthma at five years [75].

Chiu et al. found a negative association between airway microbial diversity and the existence of sensitisation to mites at early ages. Dysbiosis may influence the presence of allergic reactions in response to exposure to allergens, which may cause susceptibility to asthma in early infancy [76].

Ta et al. studied a longitudinal cohort of children in Singapore, analysing the establishment of the microbiota in the nose during the first 18 months after birth. In healthy children they found a progressive increase in diversity, with early colonisation by bacteria of the *Staphilococcaceae* (*Firmicutes phylum*) family and an increase in *Corynebacteriaceae* (*Actinobacteria phylum*). In children with rhinitis or wheezing they noted less diversity and a greater numbers of the bacteria families *Oxalobacteraceae* (*Proteobacteria phylum*) and *Aerococcaceae* (*Firmicutes phylum*). They found that being male, delivery by caesarean section, the presence of siblings and attending day-care to be determining factors in the establishment of the nasal microbiota and the cause of dysbiosis [77].

Teo et al. analysed the relationship between the nasopharyngeal microbiome, the existence of respiratory infections and early allergic sensitisation in 244 children for the first five years of life, finding that in children with early allergic sensitisation and upper airway colonisation by *Moraxella*, *Streptococcus* and *Haemophilus* the risk of chronic wheeze at five years of age increased [79], corroborating the finding of Bisgaard et al. from ten years before [74].

Depner et al. analysed the microbiota of 327 samples from the throat and 68 nasal samples from school-age children, both from farms and not from farms, by means of 454-pyrosequencing of the bacterial 16S ribosomal RNA gene. They found an association of asthma with the presence of altered nasal microbiota, characterised by a lower α and β-diversity and the abundance of *Moraxella*, above all in children who did not live on farms [80].

Dzidic et al. determined the bacterial composition in saliva samples gathered longitudinally at 3, 6, 12, and 24 months, and 7 years and found that those children who developed asthma had lower diversity together with very divergent composition at seven years, with an increase in the abundance of *Gemella haemolysans* [81].

Thorsen et al. examined the respiratory pathways microbiota in the first three months of life by through sequencing amplicons of the 16S rRNA gene in a Danish population-based cohort consisting of 700 children. *Veillonella* and *Prevotella* relative abundance and microbial diversity in the airways at one month of age was associated with asthma at six years. A relative increase of these bacteria was also associated with a reduction in TNF-α and IL-1β and an increase in CCL2 and CCL17, which in turn is an independent predictive factor for asthma. These results suggest a microbiota-immune interaction mechanism in early infancy that predisposes to infant asthma [82]. 

Powell et al. studied the temporal association of the development of oropharyngeal microbiota, determined by means of sequencing the V3–V5 region of the rRNA 16S encoding gene, with wheezing during the first years after birth in a group of 159 infants born in London and followed for 24 months. They observed a significant increase in the abundance of *Neisseria* between nine and 24 months of life in the infants who developed wheezing, whilst in those who did not wheeze there was a significant increase in the abundance of *Granulicatella* between nine and 12 months, and of *Prevotella* after 18 months, showing the existence of a temporal association between the respiratory commensal [83].

Zhang et al. studied sputum samples from 74 infants under the age of six months admitted to hospital for a first episode of bronchiolitis by respiratory syncytial virus, finding an association between the abundance of *Moraxella* and *Haemophilus*, which would modulate the inflammatory response of the airway during the episode (IL6, IL10, CXCL8), and the development of recurrent episodes of wheezing at three years of age [84].

Mansbach et al. carried out a multicentre study in the USA to examine the relationship between nasal microbiota, determined by means of sequencing 16S rRNA, at the moment of hospitalisation due to bronchiolitis in 842 infants, and in three subsequent moments, with the risk of recurrent wheezing at three years of age. They found that an increase in the relative abundance of *Moraxella* or *Streptococcus* three weeks after the first day of hospitalisation and of *Streptococcus* in the following summer was associated to a greater risk of recurrent wheezing [85].

Tang et al. analysed nasopharyngeal samples in 285 infants and found that a microbiome with a predominance of *Staphylococcus* in the first six months of life, as well as the predominance of *Moraxella* in the presence of infection by rhinovirus in the first two years of life, increased the risk of recurrent wheezing at three years and of asthma that persisted throughout infancy [87]. Davis et al., also in an American population, using data from NAHNES 2001-4 found an association between colonisation by *Staphylococcus aureus* with diagnosis and other asthma results [99]. 

The genera *Dolosigranulum* and *Prevotella* probably have a protective effect against asthma, whilst the enrichment of the genus *Streptococcus* and of the phyla *Proteobacteria* and *Firmicutes* in asthmatics probably influences the development of asthma and its severity [100]. 

These findings suggest that the upper airway microbiome could help to predict the development of asthma at older ages in life and the possibility of preventing it [13].

## 6. Microbial Dysbiosis and the Persistence, Phenotypes, and Severity of Asthma

### 6.1. Microbiome and Persistence of Asthma

Dysbiotic communities can substantially contribute to the course and severity of asthma, a relationship is observed between airway microbial dysbiosis and progression, exacerbations and response to treatment of the disease [101,102,103].

Huang et al. found that a greater abundance of *Proteobacteria* was related to worse asthma control and exacerbations due to the induction of Th17-related genes [104].

Yang et al. studied 111 patients with chronic rhinosinusitis, of whom 46 had associated asthma. They found that the patients who frequently visited the hospital emergency departments due to an asthma exacerbation tended to have a greater relative abundance of *Proteobacteria* [105].

Fazlollahi et al. performed the sequencing of ribosomal 16S RNA in nasal swabs, comparing patients with exacerbated asthma [20], non-exacerbated asthma [31] and healthy controls [21]. They found a greater abundance of some taxa such as *Prevotella buccalis*, *Dialister invisus*, *Gardnerella vaginalis* and *Alkanindiges hongkongensis* in patients with exacerbations [106]. 

Zhou et al. found that the nasal microbiota dominated by *Corynebacterium* and *Dolosigranulum* in children may reduce the loss of asthma control, in comparison with those dominated by *Moraxella*, *Staphylococcus* and *Streptococcus* [101].

McCauley et al. analysed 3122 samples of nasal secretions from 413 asthmatic patients aged 6–17 years. The predominance of the genus Moraxella was associated with an increase in exacerbations and eosinophil activation. It was possible in vitro to induce epithelial damage and the expression of IL-33 and IL-8 inflammatory cytokines [107]. Those authors recently found, in nasal samples of a paediatric population, that the seasonal dynamics of the microbiome and its transcriptome interaction was related to the exacerbations, especially in autumn [108].

Ultimately, the most common changes in the lung microbiota in asthmatics are related with a dysbiosis that favours the growth conditions of Proteobacteria, particularly *Moraxella* and *Haemophilus*, which may provoke the activation of Th2 inflammation [19].

Thorsen et al. carried out an ECA in 68 infants aged 12 to 36 months with recurrent wheezing to analyse the influence of the microbiota in the response to azithromycin. They gathered 139 hypopharyngeal aspirates prior to randomisation to azithromycin or placebo. They found that an increase in bacteria richness before the treatment was associated with the duration of the wheezing episode. Furthermore, the richness of the microbiota prior to treatment was also associated with a greater effect of azithromycin. These results suggest that the microbiota may modulate the effect of azithromycin, which supports the idea that the relationship between the airway microbiota and antibiotics is bidirectional and probably more complex than believed [109]. 

### 6.2. Microbiome and Asthma Phenotypes 

The airway microbiome is altered in chronic asthma and differs to immune response models, especially at T2 inflammation levels [20]. Clinical studies have shown an association between lung microbiota alterations and several asthma phenotypes [12]. A reduction in bacterial diversity could have an influence on the inflammatory phenotypes of asthma [54,110].

Lee-Sarwar et al. analysed the microbiome profile of rRNA 16 s and faecal metabolomics in stool samples gathered from mothers in the third semester of pregnancy and from their children at the ages of 3–6 months, 1 and 3 years, to identify the associations between the characteristics of prenatal faecal microbiome and from the first years of life and infant phenotypes of asthma (early, transitory, or active). They found that seven taxa from the maternal and two from the infant faecal microbiome were strongly associated with at least one asthma phenotype, and a longitudinal profile of the gut microenvironment was associated with early asthma, appearing at three years of life, with reduced *Bacteroides* and microbial sphingolipids in caesarean deliveries [111]

Pérez-Losada et al. studied the nasal microbiota from 163 children with different asthma phenotypes. A common core of genera was present in at least 95% of the children analysed, including *Moraxella*, *Haemophilus*, *Staphylococci* and *Streptococcus*. However, the phyla *Proteobacterias*, *Actinobacterias* and *Bacteroidetes*, and the genera *Corynebacterium*, *Dolosigranulum* and *Prevotella* varied considerably among the asthma phenotype groups, demonstrating that children and adolescents with different clinical characteristics of asthma also showed different nasal bacterial profiles, which is indicative of different phenotypes of the disease [112].

Eosinophilic asthma is the result of the activation of the Th2 response that leads to eosinophil infiltration into the airways. Here, in contrast with the neutrophil phenotype, the role of the microbiota is more heterogenous [103,113]. Eosinophil infiltration into the bronchial tissue is associated with a low bacterial load and diversity, with a negative correlation between Proteobacteria and Firmicutes and lung eosinophilia [114].

*Staphylococcus aureus* manipulates the immunology of the airway mucosa at several levels through its proteins, liberating IL-33 from the respiratory epithelium and provoking the activation of innate lymphoid cells (ILC), the liberation of type 2 cytokines by those ILC and T helper (Th) 2 cells, degranulation of mastocytes, the massive activation of local B cells and the formation of IgE and, finally, the attraction of eosinophils with the consequent liberation of extracellular traps, which adds to the epithelial damage and contributes to the persistence, exacerbations, and severity of the disease [115,116,117].

Patients with severe neutrophilic asthma present a lower microbial diversity in sputum than patients with eosinophilic inflammation, with a higher prevalence of potentially pathogenic organisms, such as *Haemophilus*, coupled with reduced commensal bacteria in the respiratory tract, such as *Streptococcus* [113,118].

*Proteobacteria* are associated with the expression of genes related with T helper (Th)17 and the inflammation driven by IL-17 may cause non-eosinophilic/non-T2 asthma that has a worse response to corticosteroids [104], whilst certain *Actinobacteria* abound in poorly controlled eosinophilic asthma [119].

Studies in individuals with neutrophilic asthma demonstrated that the microbiota composition is different: bacteria such as *M. catarrhalis*, *Haemophilus* spp. and *Streptococcus* spp. predominate. Their presence of those bacteria is related to severe bronchial obstruction, longer duration of symptoms, and neutrophilic infiltration, possibly through a mechanism driven by Th-17 [54,110,120,121]. In the most symptomatic patients, the enrichening of the airways with members of *Proteobacteria* correlated with IL-8 levels, a neutrophilic pro-inflammatory marker. Curiously, poor response to corticosteroid treatment occurs in neutrophilic asthma. In patients who are resistant to corticosteroids, an excessive growth of *Haemophilus parainfluenzae* has been observed. Moreover, *Haemophilus influenzae* and *Tropheryma* have been reported in sputum samples of patients with poorly-controlled severe cases [54,119]. Yang et al., using a long-term colonisation model of the airways by *H. influenzae* demonstrated the conversion of a model of sensitive eosinophilic inflammation to Th2 steroids in a form of neutrophilic disease that was TH17 steroid-resistant [122]. Given that inhaled steroids are a foundation of the therapy, the implication of certain bacterial genera in the resistance mechanisms may explain the cause of asthma that does not respond to treatment.

Abdel-Aziz et al. performed a longitudinal multicentre cohort study with 100 patients who had severe asthma with the aim of identifying phenotypes based on the sputum microbiome profiles and assess their stability after 12 to 18 months. They identified two conglomerates of microbiomes that revealed two distinct robust phenotypes of severe asthma that showed relative stability over time, suggesting that sputum microbiome may serve as a biomarker to better characterise asthma phenotypes [118].

Li et al. examined the relationship between sputum microbiota, asthma severity and the type of inflammation in 49 non-smoker asthmatics (25 severe and 24 non-severe) and 15 healthy subjects. They found that *Pseudomonadaceae* and *Enterobacteriaceae* were the most abundant in the severe asthmatics in comparison with the non-severe asthmatics and *Actinomycetaceae* were especially abundant in the patients with eosinophilic asthma compared to the non-eosinophilic asthmatics. The principal components analysis confirmed the positive association of the abundance of *Actinomycetaceae* and *Enterobacteriaceae* with eosinophilic asthma [123].

### 6.3. Microbiome and Asthma severity

Dysbiosis may aggravate asthma as the number of Treg cells decreases and the number of Th2 and Th17 cells increases [58]. In adult asthmatics the respiratory microbiome shows less diversity and increased abundance, above all of *Phylum proteobacteria*, particularly of the genus *Haemophilus* correlating with asthma severity [19,49], and especially the enrichment by *Moraxella*, *Streptococcus* and *Haemophilus* was associated with severe airway obstruction and neutrophilic inflammation of the respiratory pathways also in small children with persistent wheezing and in adults with severe asthma [113,124,125].

Zhang et al. studied 56 patients with stable severe asthma by means of sputum analysis and high-resolution CT scanning. The authors found that the prevalence of *H. influenzae*, *Pseudomonas aeruginosa* and *S. aureus* in the lower airways of severe asthmatics was associated with asthma persistence and exacerbations, but not with bronchial remodelling [126]. They subsequently analysed whether the microbiota in induced sputum from the lower airways was related with measures of asthma severity in 26 people with severe asthma, 18 with non-severe asthma, and 12 healthy subjects. They found that *Bacteroidetes* and *Fusobacteria* were lower in the non-severe and severe asthmatic groups. Furthermore, proteobacteria were more often found in the non-severe asthmatics compared to the controls and *Firmicutes* were increased in the severe asthmatics in comparison with the controls, characterised by the presence of *Streptococcus* spp. with eosinophilia [102].

Davis et al. found an association between nasal colonisation by *S. aureus* and an increase in asthma severity [99]. An in vitro study in asthmatic adults demonstrated that *Haemophilus influenzae* was able to activate Toll-like 4 receptors with the consequent transcription of pro-inflammatory factors such as IL-8 and also the inhibition of corticosteroid-related pathways, and as a consequence the induction of corticosteroid resistance [127]. In addition, the most severe patients, with greater bronchial obstruction, required higher doses of inhaled corticosteroids and even oral corticosteroids that alter their lung microbiome, with enrichment by potentially more pathogenic species such as *Haemophilus*, contributing to the lack of response [114].

Losol et al. recently published a systematic review in which they assessed the association between upper airway microbiota and asthma in children and adults. They found a higher α-diversity in infants aged 1–24 months with wheezing and asthma. The microbial composition of the upper respiratory tract varied a great deal at that age and *Proteobacteria (Moraxella*, *Haemophilus*, *Neisseria)* and *Firmicutes (Staphylococcus*, *Streptococcus)* were the most prevalent phyla in the children with asthma. The most abundant microbiota in adult asthmatics was *Proteobacterias*. Less consistent shifts were observed in the phyla *Bacteroidetes* and *Actinobacteria* in both age groups [128].

Goldman et al., in bronchoalveolar lavage samples obtained in bronchoscopy studies from 15 children with severe asthma, 5 with cystic fibrosis and 11 with normal bronchoscopy findings, found a significant increment in *Bacteroides* in severe asthma with respect to the controls [129].

Chun et al. carried out a systematic and integrating study of the nasal and bronchial microbiomes and the transcriptomes of the nasal and bronchial host from 27 children with severe persistent asthma and from 27 healthy controls. They found that the microbiomes and the host transcriptomes of the asthmatic children differed depending on the location (nasal or bronchial). Among the asthmatic children, *Moraxella* and *Alloiococcus* were central genera in the nasal microbiome, whilst there were no central genera among the bronchial genera. Bronchial *Actinomyces* was negatively associated with bronchial inflammation genes, which suggests that it may have a protective role. Compared to the healthy children, the asthmatic children expressed more nasal genes for ciliary function and hosted more nasal *Streptococcus*. Additionally, the nasal genera such as *Corynebacterium* negatively associated with a significantly higher number of nasal genes for inflammation in healthy children as opposed to the asthmatics. This suggests a potentially more protective role for said nasal genes in healthy children compared to asthmatics. This type of systematic and integrating study provides a window into the host-microbiome association in asthma [130]. 

All these studies point to the microbiota being a key modulator of immune, metabolic and cell functions that responds to inflammatory signals associated with asthma and probably mediates in the susceptibility, severity, and phenotype of asthma [12].

Millares et al. described the bacterial composition in the mucosa and bronchial secretions of 13 patients with severe asthma on inhaled corticosteroids through amplification and sequencing of the 16S rRNA gene, from biopsy and bronchial aspirate (BB, BA) samples. They found that *Bacteroidetes*, *Firmicutes*, *Proteobacteria* and *Actinobacteria* showed relative abundance (RA) above 5% in the BB, a limit that was attained by *Streptococcus* and *Prevotella* at genus level and *Legionella* in a lower amount. In the BA they found a higher RA of *Fusobacteria* than in the BB, whilst the RA of *Proteobacteria* was lower in the BA, also of the genus *Legionella*. The differences between the microbial communities in the BA and BB was confirmed by the beta diversity analysis and the functional analysis also showed statistically significant differences between both sample types in the metabolism pathways, cell processes, human diseases, the systems of the organism and the processing of genetic information [131]. 

**Figure 4:**
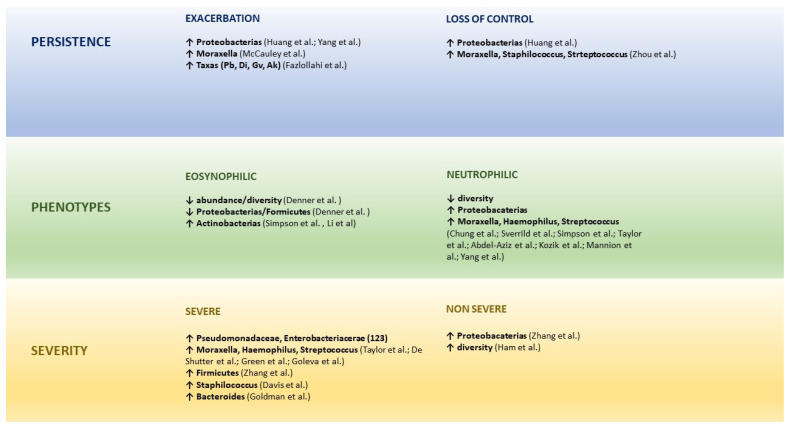
Main studies on intestinal or respiratory microbiome and asthma in children, adolescent and adult patients. Pb: *Prevotella buccalis*; Di: *Dialister invisus*; Gv: *Gardnerella vaginalis*; Ak: *Alkanindiges hongkongensis*. Abdel-Aziz et al. [118]; Chung et al. [54]; Davis et al. [99]; De Shutter et al. [124]; Denner et al. [114]; Fazlollahi [106]; Goldman et al. [129]; Goleva et al. [127]; Green et al. [125]; Ham et al. [132]; Huang et al. [104]; Kozik et al. [120]; Li et al. [123]; Mannion et al. [121]; McCauley [107]; Simpson et al. [119]; Sverrild et al. [110]; Taylor et al. [113]; Yang et al. [105]; Yang et al. [122]; Zhang et al. [102]; Zhou et al. [101].

Ham et al. studied 23 healthy individuals, 42 individuals with mild-to-severe asthma and 32 individuals with severe asthma to study the relationship between the lung microbiome, innate lymphoid cells (ILC) and asthma. Severe asthma was associated with a lower number of (NCR)+ILC3 natural cytotoxicity receptors in the lung. Similar changes were not observed in other ILC, macrophage, and monocyte subsets. Asthmatics were similar to healthy controls with regard to the alfa and beta diversity of the gut and lung microbiomes. Lung function, on the other hand, was positively associated with both the NCR^+^ILC3 frequencies as well as with the lung microbial diversity. The NCR^+^ILC3 frequencies in sputum were associated positively with the lung microbiome in the HC, but this relationship was negative in severe asthma. Taken together, these data suggest that NCR+ILC3s in the lungs may help to have a healthy bacterial airway diversity and lung function [132]. Figure 4 summarizes the results of the main studies on intestinal or respiratory microbiome and asthma in children, adolescent and adult patients.

## 7. Prevention and Treatment of Microbial Dysbiosis

**Figure 5:**
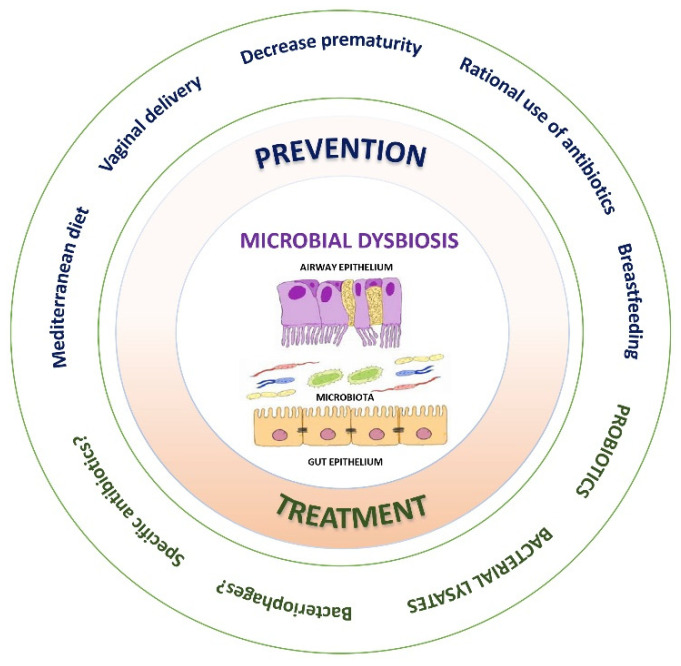
Strategies for prevention and/or treatment of microbial dysbiosis.

Preventive and/or therapeutic strategies against microbial dysbiosis are summarised in Figure 5. Identification of the environmental and host risk factors that can potentially be modified regarding the development of asthma seems to be the cornerstone to change the paradigm from treating the disease towards the primary prevention of asthma, focusing on maternal diet and nutritional supplements (high-fibre diet during pregnancy, Vit D, antioxidants, fish oil, etc), avoiding unnecessary caesarean sections, promoting breastfeeding, rational use of antibiotics, controlling severe respiratory infections in the neonatal period (RSV and rhinovirus), boosting the “farm effect”, adequate control of maternal asthma, among others [133]. The early introduction of allergic foods to train the immune system against allergic sensitisation may also be a strategy for the prevention of asthma [55]. A recent population-based cohort study carried out in Canada found an absolute reduction of 7.1/1000 in new diagnoses of asthma in children aged 1–4 years—a 26% reduction—associated with a decrease in the use of antibiotics in the first year of life and an increase in the α-diversity of the gut microbiome. 

### 7.1. Probiotics

According to the WHO, probiotics are live microorganisms, mainly lactic acid bacteria, which when administered in adequate amounts confer a health benefit. These microorganisms act on the maturation of the gut barrier and dendritic cells that influence the local and systemic immune response. Moreover, they produced substances such as SCFA, especially butyrate, which supressed the allergic inflammation of the airway. Its benefit for the prevention and treatment of asthma is still a matter of discussion.

Chen et al., in children aged 6–12 years with asthma and rhinitis, found a significant decrease in symptoms and improvement in lung function after eight weeks of *Lactobacillus gasseri* administration [134].

Huang et al. administered *Lactobacillus* to asthmatic patients aged 6–18 years for three months, and found an improvement in control, increase in the peak expiratory flow, decreased levels of IgE and decreased asthma severity [135].

Cabana et al. administered *Lactobacillus rhamnosus* to infants with a high asthma risk for the first six months of life without finding any differences in the development of asthma at five years age [136].

A metanalysis carried out by Du et al. showed that the administration of *Lactobacillus rhamnosus* would result in a reduction in the appearance of asthma (RR 0.75 [95%CI, 0.57–0.99]; I² = 11%; *p* = 0.04) [137].

### 7.2. Bacterial Lysates

The use of bacterial lysates that includes the species described as causing microbial dysbiosis and related with the development of wheezing and asthma, such as *Moraxella catarrhalis*, *Streptococcus pneumoniae* and *Haemophilus influence*, is gaining momentum, due to their non-specific immunomodulatory effect.

Emeryk et al. administered a polyvalent bacterial lysate by via sublingual 10 days per month for 12 weeks, followed up for 36 weeks, to patients aged 6–16 years with allergic asthma and found a reduction in the number of exacerbations (1.1 ± 1.3 vs. 1.9 ± 2.0, *p* = 0.01), a 55% increase in the time until the second exacerbation (hazard ratio [HR] = 0.45; 95% confidence interval [CI]: 0.27 a 0.77; *p* = 0.002) and a 74% increase until the third exacerbation (HR = 0.26; 95%CI: 0.12 a 0.58; *p* < 0.001), changing the panel of lymphocytes [138,139]. 

Recently, Nieto et al. studied the effectiveness and safety of sublingual mucosal immunotherapy with a bacterial lysate—MV130—to prevent wheezing episodes in infants under the age of three years who had suffered at least three episodes in the previous year. They excluded those infants with positive pneumoallergen skin tests. They administered MV130 or placebo to a cohort of 120 infants for six months, finding a significant drop in the number of wheezing episodes (3.0 vs. 5.0, *p* < 0.001), with a decrease in the duration of said episodes, the symptoms and the need for medication, without reporting any side effects [140].

These bacterial components reduce respiratory tract infections and may influence asthma by means of an immunoregulatory mechanism in the gut and by means of the gut-lung axis to reduce lung inflammation and hyperreactivity [19].

## 8. Clinical Implications and Future Perspectives

The major clinical implication for avoiding microbial dysbiosis involves enhancing preventive strategies, such as optimising indications for caesarean delivery and promoting breastfeeding, the rational use of antibiotics [141] and appropriate dietary interventions for mother and child, amongst others. See Figure 4 and Figure 5 for a summary of the clinical implications of dysbiosis on asthma and the strategies to deal with them. 

There is a clear need to move forward in the knowledge on microbial dysbiosis and the specific mechanisms through which the gut-lung axis, and its relationship with the origin and persistence of asthma in order to determine if that is a cause or only a finding associated to its development. In that case there would be a need to be able to design early interventions in the era of precision medicine which could have modifying properties and a positive impact on asthma trajectory [142]. Another aspect to be studied is the role of viral infection [143,144] and/or fungal dysbiosis on the effect microbial dysbiosis has on immune homeostasis [145]. 

This could be a window of opportunity to transform the conformation of the gut microbiome and of the airway or direct ourselves to specific host pathways affected by the microbiome to prevent asthma from developing or to improve its health outcomes. If we knew the relevant microbial targets, we could study the role of different strategies such as specific antibiotherapy or diet as a non-pharmacological factor. The latter should be taken into account in the management of asthma and we should even incorporate nutrition experts into our multidisciplinary units [23].

We must continue to clarify the role of probiotics, which have already been accepted and are used during or following antibiotic treatment, with the aim of a possible recolonisation. Some studies in children with allergic asthma analysing the effect of the administration of different probiotics for short periods of time have shown positive results [134,146], but a meta-analysis showed no effect except for those infants with atopic disease [147]. Although there is a consistent body of experimental evidence on the relationship between microbiota and the immune response, the use of probiotics as a therapy for asthma is still inconclusive: it should be further studied in more specific asthma phenotypes, specifically in atopic asthma, considering that different bacteria might have diverse effects. Nevertheless, this matter requires further in-depth study, not only in individuals with asthma or risk factors of asthma, but also in patients at risk of asthma plus microbial dysbiosis, to clarify the preventive effect of probiotics and their capacity to modulate the epithelial barrier function and its mode of interacting with the immune system [148], without forgetting the role that bacterial lysates may have in the near future. 

Thus, a new field of research focused on the interactions between respiratory epithelial cells, bacteria, and phages, and connecting their results with the physiopathology of asthma is starting. These new research lines propose the possibility of controlling dysbiotic bacterial populations in asthma through interventions with phages. New treatment modes for asthma could include directed and personalised recolonisation of the upper airways with phages against key bacteria. Isolating and characterising natural phages against *M. catarrhalis*, *S. aureus*, *S. pneumoniae* and *H. influenzae* may have a major importance in this context [149].

Relatively little is known about the interactions between the microbiome and the transcriptome of the host respiratory pathways in asthma. Given that asthma affects all the respiratory pathway and is affected by it, the combined study of the upper (e.g., nasal) and lower (e.g., bronchial) airways represents a powerful field of research for understanding asthma [130]. Furthermore, we should broaden the knowledge of other factors that could affect the microbiome in asthmatic patients, such as treatment with inhaled corticosteroids and the existence of comorbidities such as obesity [20].

In short, as Ver Heul et al. stated, better knowledge of the mechanisms by which microbial exposure and colonisation produce their effects on asthma is needed. Firstly, coordination within the clinical and scientific community is needed to standardise and sequencing data sharing. Secondly, techniques need to be introduced for the proper identification, isolation and characterisation of the bacterial species that modify asthma, which will enable us to develop new approaches to study and manipulate the microbiota in asthma. Thirdly, clinical and pre-clinical capacity to demonstrate the causal relationship among the microbiota must be developed. Perhaps even more important is the new insight into microbial ecology in order to implement new treatment approaches which could prevent and treat asthma [12]. 

## Data Availability

Not applicable.
